# Genome-wide SNP discovery from a pooled sample of accessions of the biofuel plant *Jatropha curcas* based on whole-transcriptome Illumina resequencing

**DOI:** 10.1186/1753-6561-5-S7-P57

**Published:** 2011-09-13

**Authors:** Orzenil B Silva-Junior, Tatiana B Rosado, Bruno G Laviola, Marilia R Pappas, Georgios J Pappas, Dario Grattapaglia

**Affiliations:** 1 EMBRAPA Genetic Resources and Biotechnology, Brazil; 2 Embrapa Agroenergy, Brazil; 3 EMBRAPA Genetic Resources and Biotechnology – Estação Parque Biológico, 70770-910, Brazilia, DF and Genomic Sciences Program - Universidade Católica de Brasília - Brazilia, Brazil

## Background

*Jatropha curcas*(JC) is an oil-rich, drought-tolerant perennial shrub of the *Euphorbiaceae* family widely dispersed throughout the world. Thought to be native to Central America, it has been the object of an increasing number of studies in recent years for it exhibits a number of appealing attributes as a promising source of biodiesel. Although its undomesticated nature and preferential outcrossed mating system would suggest a high degree of genetic variation to be exploited in breeding, studies have shown limited genetic diversity in the existing germplasm collections [1]. In spite of the increased interest in this bioenergy plant, challenges still exist to turn this species into a genuine crop and improved varieties that consolidate desirable traits are not yet available, making JC large scale plantation an uncertain business [2]. Genomic studies to potentially assist JC breeding efforts have started in the last few years. JC is diploid (2n=22), with a haploid genome size estimated at 416 Mbp [3]. EST databases focusing on gene discovery were constructed [4]and a draft genome sequence was recently published covering 285 Mbp (~68%) of the genome in 120,586 contigs with 40,929 predicted gene models [5]. The focus of our work with JC is to provide effective tools to accelerate breeding through Genomic Selection (GS) [6] and to help assess the levels, organization and enrichment strategies of genetic diversity in germplasm banks and breeding populations. To this end we have started the development of SNP markers. Available EST databases built from single individual plants do not provide the necessary sequence diversity for SNP discovery.In this work we report on the discovery of a set of SNPs for JC derived from a pool of genetically diverse accessions using Illumina sequencing and a SNP selection pipeline recently described [7].

## Methods

Genetic diversity data was used to select twelve JC accessions that maximized genetic diversity out of a germplasm collection currently serving as the foundation of a breeding program [1]. Total RNA of young expanding leaves was extracted from each individual plant and a pool of equimolar quantities of RNA was prepared. Two Illumina GAIIx single end lanes were sequenced following standard protocols. Raw reads were processed and aligned on the mapped reference genome using GSNAP [9]. GATK Unified Genotyper [10] was used to estimate the allele frequency in the pooled samples and to provide an accurate posterior probability of there being a segregating variant allele at each locus using a Bayesian genotype likelihood model. SNPs were then specifically selected to design Illumina Golden Gate Genotyping Technology (GGGT) assays based on an *in silico* estimated minor allele frequency MAF >0.10 and at least 60 bases available on each SNP flank with no additional SNPs following a procedure described earlier [7].

## Results and discussion

**Figure 1:**
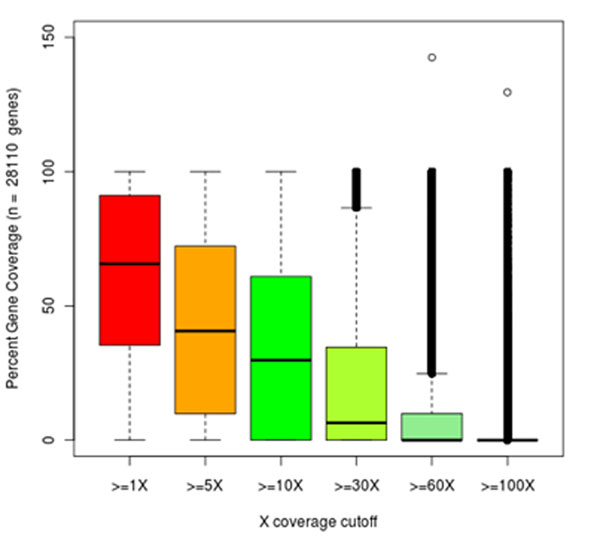
**Distribution of percent gene coverage.** Distribution of percent gene coverage attained by the transcriptome sequencing at various coverage depth levels using dereplicated reads.

The two lanes yielded a total of 74 million reads from which 66.5 million were filtered providing 11.8 Giga bases of high quality sequence. Upon mapping on the JC draft genome sequence 28,110 unigenes were sampled covering 22.1 Mbp of the 39.7 Mbp total unigene length, i.e. 56% of the transcribed portion currently predicted in the draft genome. From the 66.5 million reads, 60.8 were aligned on the genome with an average coverage of 152X of the unigene sampled. A large percentage of these reads (73%) were identical, derived either from abundant transcripts or more likely from amplification bias introduced by the PCR enrichment step during library preparation a standard occurrence in NGS [8]. After removing read amplification bias a total of 16.4 million de-replicated reads were aligned providing an average coverage of 26X of the sampled unigenes and a much more reliable substrate for SNP discovery. The distribution of percent coverage levels attained for each sampled gene was estimated (Figure 1). If a gene was completely covered by reads from the first base to the last at a depth >1X, then this gene was given a value of 100%. With no MAF and flanking sequences filtering a total of 18,225 SNPs were detected.When a MAF>0.10 was applied, 9,164 SNPs in 2,907 genes survived. When 60 bases with no SNPs on both flanks were required 1,574 high quality SNPs were recovered sampling 895 genes while 561 SNPs had coordinates not falling into any predicted gene model. These results corroborate the low level of sequence polymorphism in the breeding material and further highlight the need to widen the current germplasm base for successful breeding [1]. While a set of 768 high quality SNPs likely to show high conversion rate with the GGGT can now be developed, alternative genotype-by-sequencing technologies might provide wider genome coverage and thus assay a larger number of sequence polymorphisms.
